# Cu/CuO Composite Track-Etched Membranes for Catalytic Decomposition of Nitrophenols and Removal of As(III)

**DOI:** 10.3390/nano10081552

**Published:** 2020-08-07

**Authors:** Anastassiya A. Mashentseva, Murat Barsbay, Maxim V. Zdorovets, Dmitriy A. Zheltov, Olgun Güven

**Affiliations:** 1 The Institute of Nuclear Physics of the Republic of Kazakhstan, Ibragimov str., 1, Almaty 050032, Kazakhstan; mzdorovets@gmail.com (M.V.Z.); zheltovda@gmail.com (D.A.Z.); 2 Engineering Profile Laboratory, L.N. Gumilyov Eurasian National University, Satpaev str., 5, Nur-Sultan 010008, Kazakhstan; 3 Department of Chemistry, Hacettepe University, 06800 Ankara, Turkey; mbarsbay@hacettepe.edu.tr (M.B.); guven@hacettepe.edu.tr (O.G.); 4 Department of Intelligent Information Technologies, Ural Federal University Named after the First President of Russia B. N. Yeltsin, Mira str. 19, 620002 Yekaterinburg, Russia

**Keywords:** composite membranes, catalytic activity, copper(II) oxide microtubes, template synthesis, arsenic removal, waste water pollutant

## Abstract

One of the promising applications of nanomaterials is to use them as catalysts and sorbents to remove toxic pollutants such as nitroaromatic compounds and heavy metal ions for environmental protection. This work reports the synthesis of Cu/CuO-deposited composite track-etched membranes through low-temperature annealing and their application in catalysis and sorption. The synthesized Cu/CuO/poly(ethylene terephthalate) (PET) composites presented efficient catalytic activity with high conversion yield in the reduction of nitro aryl compounds to their corresponding amino derivatives. It has been found that increasing the time of annealing raises the ratio of the copper(II) oxide (CuO) tenorite phase in the structure, which leads to a significant increase in the catalytic activity of the composites. The samples presented maximum catalytic activity after 5 h of annealing, where the ratio of CuO phase and the degree of crystallinity were 64.3% and 62.7%, respectively. The catalytic activity of pristine and annealed composites was tested in the reduction of 4-nitroaniline and was shown to remain practically unchanged for five consecutive test cycles. Composites annealed at 140 °C were also tested for their capacity to absorb arsenic(III) ions in cross-flow mode. It was observed that the sorption capacity of composite membranes increased by 48.7% compared to the pristine sample and reached its maximum after 10 h of annealing, then gradually decreased by 24% with further annealing.

## 1. Introduction

Nanostructured materials based on copper(II) oxide (CuO) have been extensively studied by scientists over the past decades in various fields of science and technology, including management of water and wastewater [1]. The unique physical and chemical properties and high reactivity of nanoscale CuO structures (nanoparticles (NPs), nanowires (NWs), nanotubes (NTs), nanoflowers, nanopallets, etc.) offer possibilities for a wide range of applications [1]. CuO nanostructures (NSs) are actively used in nanosensors for detecting various species [2,3,4,5,6]. They are also used in the development of nanodevices for nanoelectronics [7,8]. In addition, they have antimicrobial properties against a wide range of pathogenic microorganisms [9], but their use in medical applications has been questioned due to their high toxicity [10,11]. One of the main applications of nanoscale structures of copper and its oxides is heterogeneous catalysis systems, and these NSs perform more efficiently compared to bulk analogs [12,13,14,15].

There are several methods for the synthesis of CuO NSs in the literature, such as hydrothermal production [16,17], microwave-mediated synthesis [18], aerosol pyrolysis [19], electrochemical deposition [20,21], etc. These methods can be divided into two groups: the first group includes methods for direct synthesis of CuO, and the second is based on the oxidation of copper nanostructures with various reagents or methods that yield an oxide form.

One of the simplest and most effective ways to synthesize CuO is to oxidize copper by thermal annealing method. This method does not require complex equipment and expensive reagents. Most of the studies on the thermal annealing of copper, including those in the nanoscale range, involve the use of fairy high temperatures reaching 800 °C. At temperatures below 400 °C, it is reported that not only the tenorite (CuO) phase, but also cuprite (Cu_2_O) and non-oxidized Cu phases are present in the structure [22,23,24]. In our previous works [25,26], we reported a thermal annealing process for the electrochemical deposition of copper, zinc, and cobalt nanowires at 200 °C. At long annealing times at 200 °C, we have seen irreversible changes in the morphology of nanotubes due to degradation of the polymer template, which makes the membrane mechanically unstable and greatly restricts its use [26].

In recent years, interest in track-etched membranes (TeMs) composites has risen in the scientific community due to the versatile and high performance of these materials in different applications [27,28,29]. We have previously shown the high potential of composite TEMs containing copper microtubules (MTs) as effective catalysts for p-nitrophenol hydrogenation and the Mannich reaction [30,31,32]. In addition, CuO NPs are reported to perform efficiently in the sorption of toxic metal ions such as As [33], Pb [34], Cd, Ni [35], etc.

Despite the aforementioned merits, the application of composite TeMs has rarely been studied for water treatment. There are a limited number of studies in the literature on the use of fixed (supported) copper/copper(II) oxide NSs for the removal of heavy metal ions from aqueous media [36,37]. In this study, we efficiently synthesized composite PET-based TeMs with embedded CuO MTs at temperatures below 150 °C through the thermal oxidation method. The effectiveness of the synthesis method aside, the resulting membranes performed very well in the catalytic reduction of a number of aromatic nitrophenol pollutants, namely, p-nitroaniline (4-NA), p-nitrophenol (4-NP), and p-nitrobenzaldehyde (4-NBA), and sorption of toxic arsenic(III) ions from aqueous media in cross-flow (filtration) mode.

## 2. Materials and Methods

### 2.1. Materials

Copper(II) sulfate pentahydrate, potassium sodium tartrate, palladium chloride, 4-NP,4-NA, 4-NBA, and sodium borohydride were purchased from Sigma-Aldrich (Schnelldorf, Germany). Certified reference solution containing 0.1 g/L of As(III) was purchased from Ecroskhim (Saint Petersburg, Russia). All other chemicals and solvents were of high purity and were used without further purification. Deionized water (18.2 MΩ/cm, Aquilon D-301) was used for the preparation of all aqueous solutions. PET Hostaphan^®^ RNK (film thickness is 12.0 microns) was purchased from Mitsubishi Polyester Film (Wiesbaden, Germany) and was irradiated by ^84^Kr^15+^ ions with 1.75 MeV/nucleon energy and 4 × 10^7^ ion/cm^2^ fluence to obtain TeMs (Cyclotron DC-60, Institute of Nuclear Physics of Kazakhstan, Nur-Sultan).

### 2.2. Electroless Synthesis of Composite Membranes

PET TeMs were used as the polymer template (the density of pores is 4∙10^7^ ion/cm^2^ and the pore diameter does not exceed 430.2 ± 6.5 nm). After the sensitization procedure (50 g/L SnCl_2_, 60 mL/L HCl (37%)), PET TeMs were activated (0.1 g/L PdCl_2_, 10 mL/l HCl (37%) for 6 min. The composition of the deposition solution: KNaC_4_H_4_O_6_ × 4H_2_O: 18 g/L; CuSO_4_ × 5H_2_O: 5 g/L; NaOH: 7 g/L, CH_2_O: 0.13 M, pH = 12.45 (H_2_SO_4_) [38]; deposition time: 40 min; temperature: 10 °C. After the completion of the deposition procedure, the samples were washed in ethanol and dried in air.

### 2.3. Thermal Annealing

The thermal annealing of the microtubules (MTs) formed in the PET template matrix was carried out in air atmosphere using SNOL-350 muffle furnace in the temperature range of 115 to 150 °C. The annealing time ranged from 1 to 24 h.

### 2.4. Composite Characterization

The pore size of the original template and the structural parameters of MTs were determined by porometry method using the Hagen–Poiseuille Equation (1) [39],

(1) $$Q=\frac{8\pi }{3MRT}\sqrt{\frac{nr^{3}\Delta p}{l}}$$

where Δ*p* is the pressure difference, MPa; *M* is the molecular mass of the gas, dyn × cm^−2^; *R* is the universal gas constant, erg/(mol × K); *n* is the number of microtubes per square centimeter of membrane area (template pore density); *l* is the membrane thickness, cm; and *T* is temperature, K.

The structure of the samples was elucidated using a JEOL JFC-7500F (Tokyo, Japan) scanning electron microscope (SEM). The elemental composition of the composites was studied by a Hitachi TM3030 SEM (Hitachi Ltd., Chiyoda, Tokyo, Japan) equipped with a Bruker XFlash MIN SVE microanalysis system (Bruker, Karlsruhe, Germany) at an accelerating voltage of 15 kV. Total scanning time during the generation of elemental mappings was 10 min. The X-ray diffraction (XRD) analysis was performed using a D8 Advance diffractometer (Bruker, Karlsruhe, Germany) in the angular 2(θ) range of 20 to 90° with a step size of 0.02° (measurement time: 1 sec; voltage: 40 kV; current: 40 mA). The average crystallite size was determined using the Scherrer Equation (2):

(2) $$\tau =\frac{k\lambda }{\beta \cos\theta }$$

where *k* is the shape factor (0.9), *λ* is 1.54 Å, *β* is the full width half maximum (FWHM), and *θ* is the Bragg angle.

The lattice parameter (α) was calculated using the Nelson–Taylor extrapolation function (3):

(3) $$\alpha =f\left[\frac{1}{2}\left(\frac{cos^{2}\theta }{sin\theta }+\frac{cos^{2}\theta }{\theta }\right)\right]$$

where *f* is the Nelson–Taylor function and *θ* is the Bragg angle. The value and the error in calculating the parameter α are determined by linear extrapolation of this function to the zero value of the argument (*θ* = 90°).

XPS measurements were made by using a Thermo Scientific K-Alpha spectrometer (Waltham, MA, USA) with a monochromatized Al Kα X-ray source (1486.6 eV photons) at a constant dwell time of 100 ms for several scans and pass energy of 30 eV with step of 0.1 eV for core scan spectra and 200 eV with step of 1.0 eV for survey scan spectra. The pressure in the analysis chamber was maintained at 2 × 10^−9^ Torr or lower. The binding energy (BE) values were referred to the C1s peak at 284.7 eV. Processing of the data was carried out by Avantage software.

The thermal stability of PET TeMs was investigated by thermogravimetric analysis (TGA) using Perkin–Elmer Pyris-1 (Waltham, MA, USA) model thermogravimetric analyzer in the temperature range of 25 to 895 °C under nitrogen atmosphere (flow rate: 20 mL/min). The heating rate was maintained at 10 °C/min.

### 2.5. Catalytic Performance of Cu/CuO/PET Composite Membrane

To study the catalytic activity of composite membranes, a 2 × 2 cm^2^ sample was immersed in a 20.0 mL reaction mixture of aromatic nitro compound (7.82 × 10^−6^ M) and NaBH_4_ (7.82 × 10^−3^ M) [40]. All experiments were carried out by continuous stirring at 25 ± 0.2 °C. The optical density was measured in the wavelength range of 200 to 600 nm using UV–Vis spectrophotometer (Specord 250 BU, Jena Analytic, Jena, Germany) every 1–2 min. The degradation rate was calculated using the following Equation (4),

(4) $$D=\frac{C_{0}-C}{C_{0}}\times 100\%=\frac{A_{0}-A}{A_{0}}\times 100\%$$

where *C* is the concentration of the nitro compound and *A* is the absorbance measured at 400 nm, 380 nm, and 277 nm for 4-NP, 4-NA, and 4-NBA, respectively, at different time periods.

All the catalytic experiments were carried out using at least three samples. In reusability experiments, each sample was tested 5 times. After each cycle the catalyst was washed with deionized water, dried, and used without additional purification or activation procedure.

### 2.6. Adsorption of As (III) in Cross-Flow Mode

The As(III) sorption experiments of the as-prepared and annealed composites in cross-flow mode were carried out using polypropylene syringe filter holder (Whatman, U.K.) and peristaltic pump, LOIP (Saint Petersburg, Russia). The temperature was 25 ± 1 °C for all filtration experiments. The effective filter holder diameter was 2.5 cm with an area of 4.9 cm^2^ for each sample and the amount of loaded copper was calculated to be 3.92 mg. Feed As(III) solution (pH 4.0) at a concentration of 10 ppm was prepared by diluting certified reference solution of As(III) ions (0.1 g/L, Ecroskhim, (Saint Petersburg, Russia). During each filtration experiment, the feed solution was spiked with As(III), filtered through the composite membranes at a constant permeate flux of 10 mL/min. Aliquots were collected at every 15 mL. Each experiment was repeated in triplicate. The concentration of As(III) in aliquots was determined by ICP-MS (Thermo Fisher Scientific, XSeries 2, (Waltham, MA, USA). The sorbed amounts were calculated by using Equation (5) [41]:

(5) $$Q_{e}=\frac{\left(C_{0}-C_{e}\right)\times V}{m}$$

where *Q*_e_ is the amount of As(III) ions sorbed onto the unit mass of copper (mg/g), *C*_0_ is the feed concentration (mg/L), *C*_e_ is the concentration of As(III) in aliquots (mg/L), *V* is the volume of solution (L), and *m* is the amount of Cu/CuO loaded on the membrane used (g).

## 3. Results

### 3.1. Synthesis and Thermal Annealing of Cu/PET Composites

Electroless template synthesis is a very flexible and versatile solution-based approach for metal thin film deposition [42,43]. In the synthesis of Cu MTs, a PET TeM template functionalized with Pd catalytic nuclei was immersed in a plating copper solution, rendering the process facile, cost-efficient, and scalable up to sample size of 10 × 15 cm. The thin layer of the Pd nuclei was used only once to initiate the plating, and was then covered by the continuous building up of the electroless copper layer [30,44]. This technique does not require complex instrumentation and the deposition process is based on the autocatalytic reduction of Cu^2+^ ions inside the plating solution at the surface of the template [45,46].

The synthesized composite Cu/PET membranes had a thickness of 12 microns, copper MT wall thickness of 75 ± 5 nm, and an average internal diameter of 280 nm, which allowed us to successfully use the composite membranes for filtration application. The thermal annealing process applied to generate CuO phase consisted of several sequential operations: heating the sample to a specified temperature, exposing it to a constant temperature for a defined period of time, and then slowly cooling the sample to room temperature. As previously shown [26], the main advantages of heat treatment are reduction of internal stress arising during production and increase of mechanical properties by reducing the number of defects and changing the defective structure; the structure becomes more ordered and resistant to external influences after heat treatment.

Prior to annealing experiments with Cu/PET membranes, the thermal stability of the PET polymer template was evaluated by means of TGA. Figure S1 presents the TGA thermogram and its derivative curve for the PET TeM. It can be seen that thermal degradation of PET starts above 430 °C and reaches to its maximum at 464.5 °C, which indicates the high thermal stability of the polymer part of the composite membrane.

**Figure 1:**
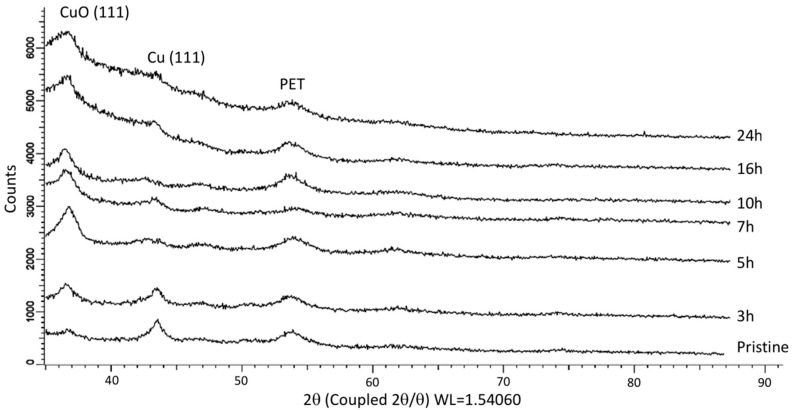
X-ray diffraction (XRD) patterns of the pristine and composite membranes annealed at 140 °C for indicated time periods.

X-ray diffraction (XRD) is obviously one of the most effective tools to study the crystal structure of materials and to confirm their purity and phase contents. As can be seen from the XRD patterns of pristine and annealed (at 140 °C) membranes in Figure 1, characteristic peaks consistent with the metallic copper, tenorite CuO phase, and PET membrane are apparent in the diffractograms. The X-ray diffractogram of pristine Cu/PET sample identifies characteristic peak (111) of the face-centered cubic (fcc) crystalline lattice of metallic copper (JPDF No. 04-0836, standard value *a* = 3.613 Ǻ) at 2*θ* equal to 43.48 degrees. The characteristic tenorite CuO peak (111) (JCPDS No. 41-0254, standard value *a* = 4.685 Ǻ) is distinguished clearly at ~36.84°, while PET contribution appears above 54° as a broad peak. The other characteristic peak of CuO at 2*θ* ~35.5° corresponding to (11–1) reflection was not detected on the XRD patterns of annealed composites as its intensity is extremely low. The formation of tenorite CuO phase in annealed samples was further confirmed by XPS analysis to be discussed later.

This section may be divided by subheadings. It should provide a concise and precise description of the experimental results, their interpretation, as well as the experimental conclusions that can be drawn.

**Figure 2:**
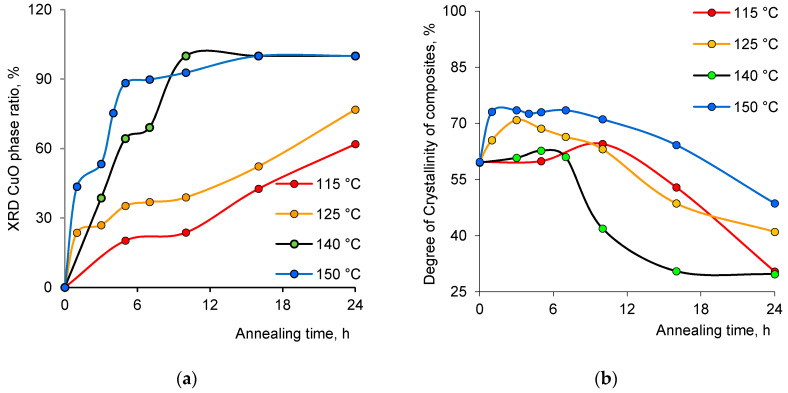
(**a**) Change of tenorite CuO phase ratio and (**b**) the degree of crystallinity (DC) of the composites depending on time and temperature of annealing.

The annealing of samples was carried out in the temperature range of 115 to 150 °C over time periods varied from 1 to 24 h. The annealing efficiency was determined by calculating the ratio of cuprite CuO phase in comparison to metallic Cu phase in the composite (Figure 2a). It should be noted that no Cu_2_O phase formation was recorded during the annealing of samples in the specified temperature range. The phase composition was determined using the Rietveld method, which is based on estimating the areas of diffraction peaks by approximating them and determining convergence with reference values for each phase [47,48]. In case of the predominance of amorphous structures such as the PET template, the phase composition is determined based on the most intense peak, which in our case is CuO (111) [49]. The volume fraction of the CuO phase was determined by using Equation (6) [50]:

(6) $$V_{\mathrm{admixture}}=\frac{RI_{\mathrm{phase}}}{I_{\mathrm{admixture}}+RI_{\mathrm{phase}}}$$

where *I*_phase_ is the average integral intensity of the main phase of the diffraction line, *I*_admixture_ is the average integral intensity of the additional phase, and *R* is the structural coefficient equal to 1. As can be seen from the data presented in Figure 2a, almost complete conversion of copper to copper(II) oxide is possible only at 140 °C and above, and ~100% tenorite phase is attained after 10 h of annealing at this temperature. A very small peak observed at 43.3° (2*θ*) in Figure 1 for the sample annealed for 10 h indicates a minor contribution of the metallic Cu phase to the composition of microtubules. The amount of metallic Cu phase was determined as 3.8% for this sample from XPS analysis, which will be discussed in further parts of this paper. It is worth noting that at a temperature of 150 °C, the polymer template is mechanically weakened after 7 h of sample processing, which makes its further use difficult in sorption and catalysis experiments. Thus, the thermal annealing mode at 140 °C was selected as the optimum condition, and the structure of the composites annealed at different time intervals at this temperature was studied in more detail.

One of the other important parameters is the degree of crystallinity (DC) of microtubules at the end of the annealing process (Figure 2b). The change in the full width at half maximum (FWHM) value of the main diffraction lines on X-ray diffraction patterns depends directly in the DC of the samples. The width of the registered FWHM lines was calculated by approximating the lines on the diffractogram with the necessary number of symmetric pseudo-Voigt functions, which, in the long run, allowed us to characterize the perfection of the crystalline structure and to evaluate the DC. Figure 2b displays the change in DC in composite TeMs as a function of annealing time at different temperatures under uniform heating conditions (in air atmosphere, under constant convection). In all cases, the crystallinity was initially found to increase slightly and then decrease at longer periods of annealing. This indicates that at longer annealing times, nanosized crystals melt and recrystallize to some extent [51,52,53]. After this melting/recrystallization process, the size, perfection, or both of the crystallites are decreased due to the fact that the recrystallization process occurs in a completely different environment than the initial synthesis condition, which leads to a decline in overall crystallinity [54]. The position and intensity of the peaks in XRD diffractograms, their corresponding interplane distances, FWHM values, as well as unit cell parameters and average crystallite sizes calculated for the original and annealed composites are summarized in Table 1.

Copper, especially in nanosize, is extremely sensitive to air and the oxide phases are thermodynamically more stable, making the formation of a surface oxide layer on copper nanoparticles inevitable [55,56,57]. A small amount of CuO (2*θ* = 36.84°) was observed in the XRD pattern of the unannealed sample, as can be seen in Figure 1 and Table 1, because the XRD analysis was carried out in the air atmosphere. The slight change in values of the crystal lattice parameter *a* compared to its standard value of 3.615 Å (according to JCPDS Cu No. 04-0836) is attributed to an increase in the oxide content of the Cu/CuO/PET composites as well as the average size of the crystallites. As can be seen from the XRD patterns presented in Figure 1, the intensity of the CuO phase is increasing with annealing time due to the increase in thermal vibrations of the CuO crystal lattice [25].

**Figure 3:**
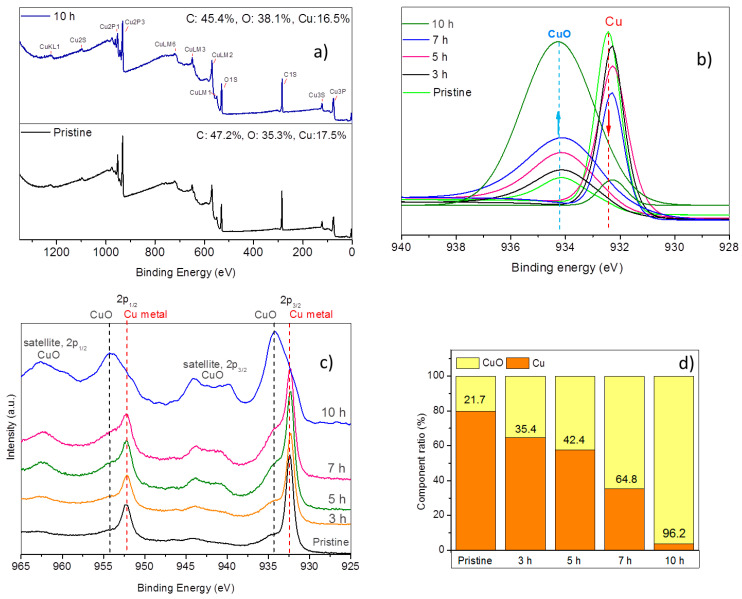
(**a**) Survey wide-scan XPS spectra of pristine unannealed membrane and 10 h annealed samples. (**b**) Cu2p core-level XPS spectra of samples prior to and following annealing for different times. (**c**) Peak components attained after curve fitting of Cu2p_3/2_ signal of the samples. (**d**) Relative ratios (%) of CuO and metallic Cu calculated from the areas of Cu2p_3/2_ peak components prior to and following the annealing.

The XPS study of pristine and annealed PET samples was carried out to evaluate the chemical changes occurring on the surface during annealing for different time periods (Figure 3).

The photoelectron peaks for carbon (C1s) and oxygen (O1s) in PET structure appear at 284.5 eV and 533.0 eV, respectively, as can be seen in Figure 3a [58]. Characteristic copper signals appeared in the survey wide-scans, proving the presence of this element in the structure. After 10 h of annealing, a slight increase in the amount of O is attributed to oxidation of copper expected to occur. In the lattice of CuO, each atom has four nearest neighbors of the other kind: Cu atoms are at the center of an O rectangle, while O atoms are at the center of a Cu distorted tetrahedron [59]. The crystallographic structure of CuO corresponds to a different electronic environment compared to copper metal. Therefore, the identification of copper in its metallic (zero-valent) and oxidized (Cu(II), CuO) states can be easily carried out by XPS. The Cu2p spectrum in CuO is characterized by high-intensity shake-up satellite peaks on the high binding energy side (~9 eV) of the main 2p_3/2_ and 2p_1/2_ photoelectron peaks [60,61]. These satellite peaks are absent in the spectra of metallic Cu. Furthermore, the main 2p_3/2_ and 2p_1/2_ peaks appear at higher binding energy in the case of CuO and are considerably broader due to shake-up process [61]. As can be seen in Figure 3b, the pristine unannealed membrane presents characteristic Cu2p_3/2_ and Cu2p_1/2_ peaks at 932.4 eV and 952.2 eV, respectively. These peaks are in a very good agreement with previously reported data [62].

The pristine sample presents additional low-intensity Cu2p_3/2_ and Cu2p_1/2_ components at higher binding energies, arising from CuO species. This shows that copper loaded to PET contains minute amount of CuO even before annealing. Here, it should be noted that the top most surface layers analyzed by XPS are more sensitive to oxidation compared to bulk. After 10 h of annealing, broad Cu2p_3/2_ and Cu2p_1/2_ peaks at higher binding energies and their corresponding satellite peaks clearly indicate CuO species in the structure [63]. Careful data analysis can assist in obtaining the relative ratios of Cu and CuO components in samples by curve fitting the Cu 2p_3/2_ peak. The peaks are generally fitted by a nonlinear least square fitting routine using a weighted sum of Gaussian and Lorentzian component curves [64]. In curve fittings we applied a combined Gaussian/Lorentzian (80%/20%) line shapes and a Shirley-type background using Kratos software. The fitted peak components of Cu 2p_3/2_ photoelectron signal are presented in Figure 3c. As can be clearly seen from this figure, the amount of copper metal in annealed samples decreases while relative amount of CuO increases by annealing time. The ratio (%) of Cu and CuO in each sample was calculated from the area under the fitted peak of the corresponding component. As it is clear from Figure 3d, the amount of metallic copper gradually decreases by annealing, and almost a complete oxidation (96.2%) is attained after an annealing time of 10 h in agreement with the above-mentioned XRD results.

**Figure 4:**
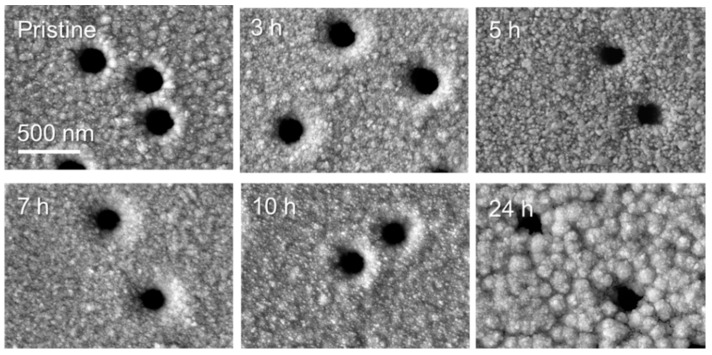
SEM images of the composite membrane after thermal annealing at 140 °C for various times.

The change in the morphology of the composite membrane surface was investigated using the SEM method. As can be seen from the electronic microphotographs shown in Figure 4, agglomeration of copper NPs takes place on the surface of the composite membrane with increasing annealing time.

**Figure 5:**
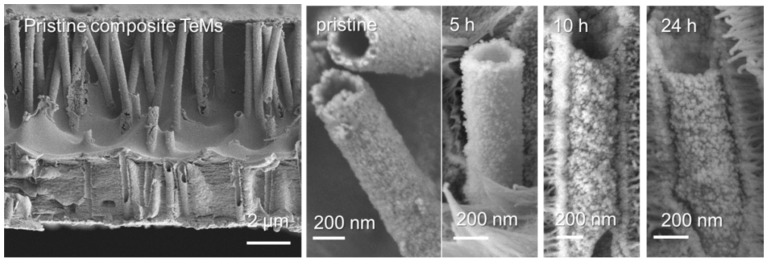
SEM images of composite membrane and individual copper microtubules attained at different annealing times.

The change in the morphology of individual MTs depending on the annealing time is shown in Figure 5. For obtaining high-quality cross-sectional images of the composite films as well as released MTs, the composite membrane was irradiated by a UV lamp for 10 days from each side. As can be seen from the presented images, no deformation of the MTs structure is observed even after 24 h of annealing at 140 °C, and the change in the surface morphology of individual microtubules is identical to the changes taking place on the composite surface indicated in Figure 4.

**Figure 6:**
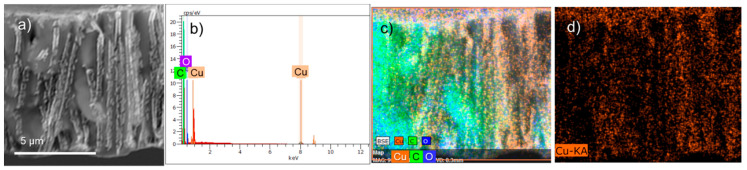
Cross-sectional SEM image (**a**); EDX spectrum (**b**); and combined EDX mappings of Cu, C, and O elements (**c**) for pristine composite membrane. Individual SEM-EDX mapping of copper element across the image (**d**).

In order to evaluate the chemical composition of the composite membrane and to verify the dispersion of copper element within the cross section of PET membrane, the energy dispersive X-ray analysis (EDX) and elemental mappings were carried out. The EDX spectrum and elemental maps acquired for the pristine composite are shown in Figure 6. Strong signals from the C and O atoms can be attributed to the PET template (Figure 6b). The individual EDX mapping of copper presented in Figure 6d reveals a uniform distribution of this element throughout the cross section of the polymer template.

### 3.2. Assessment of Catalytic Activity

**Figure 7:**
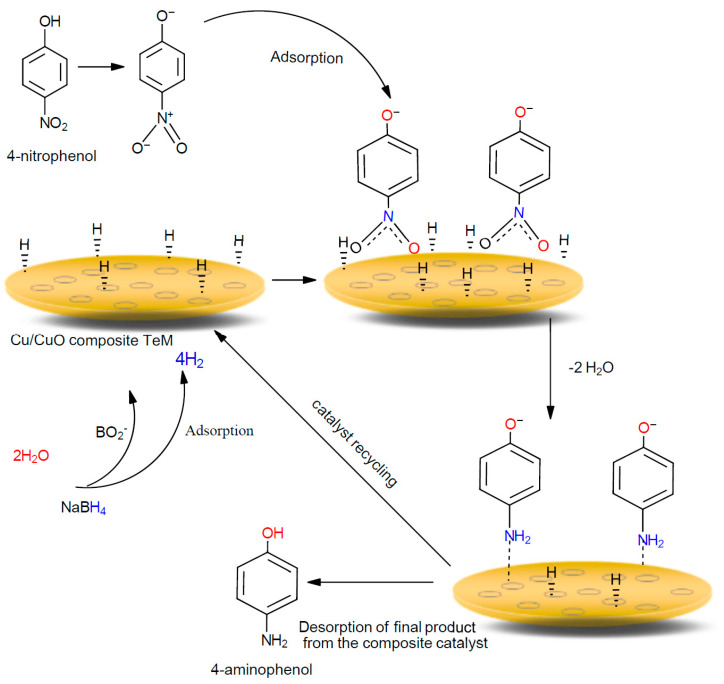
Schematic representation of 4-NP reduction on the surface of Cu/CuO composite TeM according to Langmuir–Hinshelwood mechanism.

The hydrogenation of nitrobenzene and its derivatives is one of the most well-known liquid phase heterogeneous catalytic processes, which finds practical application in the production of organic pigments, biologically active substances, pharmaceutical substances, additives to polymer materials and motor fuels, etc. [65,66]. It is no accident that highly efficient, environmentally friendly liquid-phase hydrogenation technologies are widely used by the chemical enterprises of leading manufacturers of fine organic synthesis products. One of the most promising methods for the synthesis of aromatic and fatty aromatic amines in both laboratory and industrial practice is the catalytic hydrogenation of nitro compounds [65]. On the other hand, nitrophenols and their derivatives resulting from the production of pesticides, insecticides, synthetic colors, and herbicides are among the most common pollutants in industrial wastewater [67,68]. Therefore, the development of effective catalysts for the decomposition of nitrophenols is an urgent and necessary task. To date, many papers have dealt with the reaction of liquid phase hydrogenation of aromatic nitro compounds by using nanoscale heterogeneous catalysts (Figure 7) [69,70,71,72,73,74]. The influence of various factors such as deposition temperature, testing mode, template modification, etc. on catalytic performance has been studied for various composite TeMs with deposited NTs of copper subgroup metals [75,76,77]. In this study, we attempted to determine the effect of thermal annealing time and thus the ratio of CuO phase on the efficiency of hydrogenation of nitroaromatic compounds in the presence of sodium borohydride (Figure 7). After each test cycle, the catalyst (CuO/PET membrane) was washed in deionized water, dried, and then used again without any activation or regeneration procedures.

The reduction of nitroaromatic compounds proceeds according to the Langmuir–Hinshelwood mechanism and was detailed in [69,78,79,80,81]. According to this mechanism, hydrogenation of nitrobenzene and its derivatives takes place through two main stages [82]. In the first stage, borohydride and nitrophenolate ions in aqueous solution are reported to be adsorbed onto the nanocatalyst surface [83]. The second stage consist of three steps: equilibrated adsorption of 4-nitrophenolate to form 4-aminophenol by addition of hydrogen species through formation of the 4-hydroxylaminophenol intermediate [84], removal of two water molecules from nitro group, and finally detachment of the 4-aminophenol molecule from the nanoparticle surface to provide place for another catalytic cycle. The reduction of 4-NP among the nitroaromatic compounds studied is schematically illustrated in Figure 7.

The reduction of nitroaromatic compounds follows pseudo-first-order kinetics, which allows one to calculate the rate constant from a change in the concentration of reactant using the following Equation (7) [85],

(7) $$\ln\left(\frac{C_{0}}{C}\right)=kt$$

where *C*_0_ is the initial concentration of the reactant in feed solution (mg/L), *C* is the concentration of the reactant at time *t* (min), and *k* is the reaction rate constant (min^−1^). The rate constant *k* is determined from the slope of the ln(C_0_/C) vs. reaction time *t* plot presented in Figure S3, and a linear relationship clearly shows that the hydrogenation of studied nitrophenols catalyzed by the composites follows a first-order reaction mechanism.

**Figure 8:**
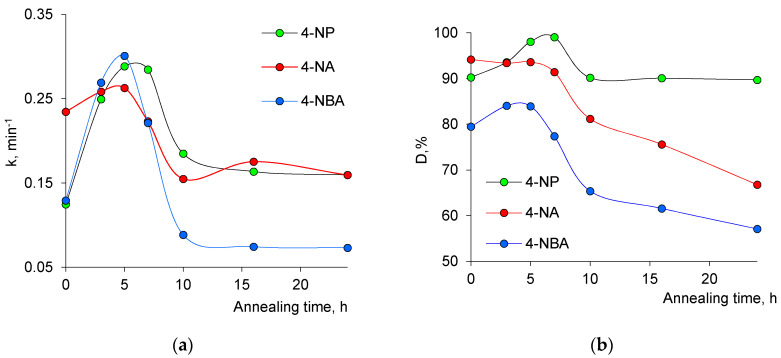
Composite track-etched membrane catalyzed reduction of aromatic nitro compounds: change of the rate constant, *k* (**a**), and degree of hydrogenation, *D* (**b**), as a function of annealing time at 140 °C.

Figure 8a,b shows the change in the reaction rate constant, *k*, and the degree of hydrogenation, *D*, depending on the thermal annealing time of the composite membrane. As seen in Figure 8a, the catalytic activity of the composites significantly increases with increasing annealing time, and thus with tenorite CuO phase ratio, up to 5 h of annealing. At the end of this annealing period when the samples reached maximum catalytic activity, the ratio of copper(II) oxide phase and the degree of crystallinity were determined as 64.3% and 62.7%, respectively. A significant reduction of more than 60% was encountered in reaction time for the 5h-annealed sample compared to the raw sample. After this first five-hour period, which is common in all three studied reactions a marked decrease in *k* and *D* values is noticed at annealing times exceeding 7 h. This decrease is assumed to be associated with the significant decrease in the degree of crystallinity (DC) encountered for annealed samples. DC values are reduced by more than 31% and 51% at the annealing times of 10 and 24 h, respectively, compared to DC at 7 h. Previously, high crystallinity has been shown to be an important feature of heterogeneous nanocatalysts that enhances catalytic properties in a wide range of reactions [86,87,88]. Thus, the increasing amount of amorphous regions in Cu/CuO composites demonstrated in Figure 1 and Figure 3 has a negative impact on the catalytic performance; a similar effect has recently been detailed in [89].

To study the effect of temperature on the degree of hydrogenation of the nitrophenols, additional tests were conducted at temperatures of 9, 13, 20, and 25 °C. As can be seen in Figure S3, the Arrhenius plot of ln *k* versus 1/T gives a straight line with a slope –*E*a/R, from which the activation energy (*E*a) was calculated. Table 2 shows the data on changes in *E*a values depending on the annealing time and the ratio of CuO phase determined from XRD data.

Obviously, for the 4-NBA hydrogenation reaction, there is a significant decrease in the activation energy of reduction with the increase in the amount of oxide phase in the composite membrane. The *E*a value of 5- and 24-hour-annealed composite decreased by 68.5 and 48%, respectively, compared to the unannealed composite for the reduction reaction of 4-NA. For the 4-NP hydrogenation reaction, the minimum *E*a value was calculated after 10 h of annealing for the sample with a CuO phase ratio of 100% (XRD). Among the aromatic nitro compounds, the highest *E*a values were determined for the reduction of 4-NBA. Furthermore, a pattern of decreasing *E*a with increasing annealing time and CuO phase ratio was observed for this compound. Table 3 compares our results with the previously published data on the catalytic activity of various nanoscale structures of copper and copper(II) oxide and relevant composites in the hydrogenation reaction of aromatic nitro compounds. It should be noted that it is rather difficult to directly compare the data of various studies as some determining parameters, such as amount of loaded catalyst, the initial concentration of aromatic nitro compounds in the tests, the type and size of catalyst, etc., are not exactly the same. Still, it can easily be said that our results compete closely with the existing alternatives and that the obtained composite membranes are promising considering particularly their practicality and high surface areas.

Stability and multiple reusability are of great importance for the practical application of catalysts. Catalysts deposited on a substrate have a number of advantages over non-fixed analogs. The non-fixed catalytically active nanoparticles or nanopowders should be carefully separated from the solution by filtration, precipitation, and centrifugation before reuse, which is a rather time-consuming and cost-ineffective procedure, and some amount of nanoscale catalyst may be lost [95]. The use of a substrate additionally increases the available metal surface area, which is directly related to the catalytic activity. Thus, the substrate not only provides the structural scaffold and stabilizes the active particles, but also increases the efficiency of the catalyst [96,97].

**Figure 9:**
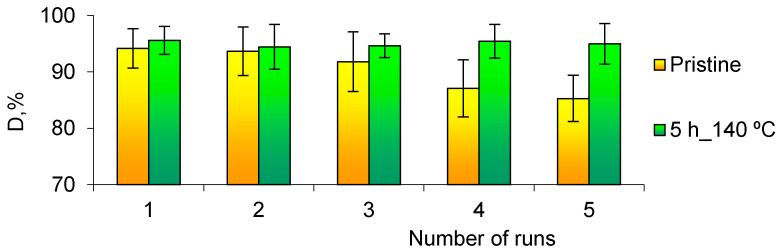
Efficiency of pristine and annealed (140 °C, 5 h) composite membranes in 5 sequential cycles of reduction of 4-NA.

Flexible composite TeMs with deposited nanotubes/nanoparticles can be easily removed from the medium after the reaction and reused without any additional activation process. We performed five consecutive test cycles to assess the stability of the catalytic properties of the studied CuO/PET composites. The results presented in Figure 9 were obtained without any additional activation and regeneration procedures after each cycle. This figure shows that upon annealing the composite membrane for 5 h, its catalytic stability is maintained at least for five consecutive uses.

As can be seen from Figure 9, the degree of hydrogenation (D) of 4-NA remains unchanged throughout the entire five test cycles for the sample with 64.3% CuO phase obtained after 5 h of annealing. For the pristine (unannealed) composite, on the other hand, the *D* value decreased by 9.5% at the end of the 5th test cycle.

The decrease in activity of the unannealed composite is most likely due to leakage of NPs from the membrane as well as decreasing of the copper crystallinity in agreement with our previously work [98]. In general, it can be concluded that thermal annealing of composite track-etched membranes with chemically deposited copper microtubules can improve the catalytic properties especially in case of reuse, and the degree of crystallinity of samples is one of the major parameters to be considered.

### 3.3. Flow-Through Removal of As(III)

Arsenic is one of the most common and dangerous toxic elements for human health and the environment [99]. In natural waters, arsenic exists mainly in the form of arsenate As(V) and arsenite As(III) [100]. According to the World Health Organization (WHO), the European Union (EU), and the United States Environmental Protection Agency (USEPA), the maximum permissible concentration for As in drinking water is 10 micrograms/L [101]. In its reports, WHO states that approximately 1 in 100 people who consume water containing more than 0.05 mg/L of arsenic over a long period of time may die from arsenic-related cancer. This rate reaches 10% in cases where As concentrations exceed 0.05 mg/L. Therefore, the removal of arsenic from water sources is one of the most important issues in relation to the protection and preservation of the environment and human health. The main processing technologies for arsenic removal include electroless deposition, membrane filtration, adsorption processes, and ion exchange [100]. There are a number of works using nanoscale materials as adsorbents of toxic arsenic compounds, such as copper oxide NPs [33,102,103,104], composites based on nickel ferrite and polyaniline [105], copper and carbon nanotubes [36], zinc sulfide NPs in the cysteine–ZnS:TiO_2_ system [106], and Fe/Cu NPs [107]; moreover, a number of review papers are available [108,109,110,111]. Thus, research aimed at preventing arsenic contamination of groundwater and/or mitigating the consequences of such contamination is very remarkable and timely. It is necessary to develop new ways to reduce the health risks associated with the arsenic-contaminated water.

**Figure 10:**
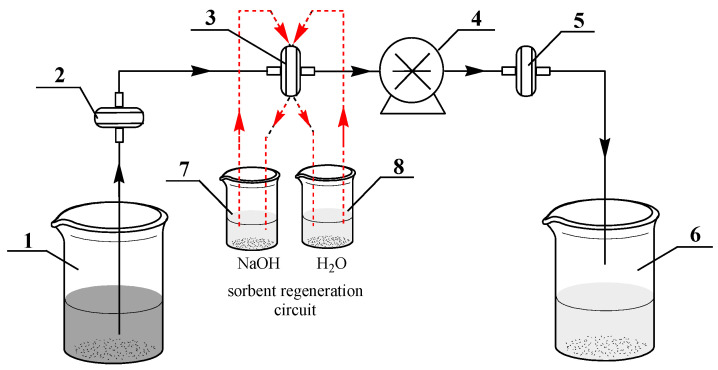
General scheme of the laboratory setup for studying the As(III) sorption performance activity of the composite TeMs. (1) As(III)-contaminated feed solution, (2) coarse prefilter, (3) membrane fine filter, (4) peristaltic pump, (5) final coarse filter, (6) container–receiver of purified water, (7) tank with regeneration solution, and (8) distilled water.

First, we studied the effect of the flow rate of arsenic(III) solution on the sorption capacity of the composites. The diagram of the assembly used for testing composite membranes is shown in Figure 10. The assembly includes a coarse input filter (2), prepared from PET TeMs with average pore diameter not exceeding 330 ± 15 nm. This input filter was used to remove various types of particulate pollutants from the feed solution (1). The pore size of the final coarse filter (5) is less than 200–250 nm and it is used to further purify the eluate from residual impurities of the feed solution (1), as well as possible trace amounts of the composite membrane (3) that may occur through physical ruptures during the filtration process.

**Figure 11:**
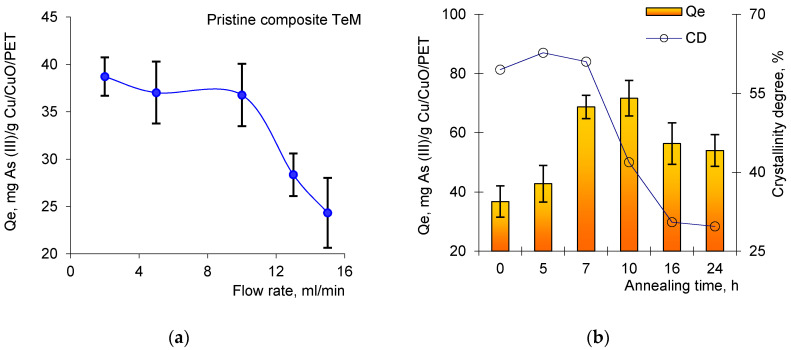
Change in As(III) sorption capacity at equilibrium (*Q*e) depending on pumping speed of working solution across the pristine composite TeMs (**a**) and *Q*e as a function of thermal annealing times at 140 °C and their corresponding degree of crystallinity (**b**).

The structure of the assembly also provides an additional filter element regeneration loop based on the composite TeMs. It is adapted to the assembly based on the data of a previous work and includes sequential washing (3) in NaOH solution (0.3 M) and distilled water. Figure 11a shows the data on changes in the sorption capacity at equilibrium (*Q*e) depending on the speed of pumping the arsenic solution through the pores of composite track-etched membrane with copper microtubules (the initial unannealed sample).

Figure 11a shows that the arsenic sorption efficiency of the pristine Cu/PET composites decreases significantly when the peristaltic pump speed is over 10 mL/min. The sorption capacity decreases slightly in the speed range of 3 to 5 mL/min, but the time required to collect aliquots increases 2–3 times in this range. Thus, all further tests were carried out at a pumping rate of 10 mL/min to speed up the process. The results in terms of As(III) sorption efficiency (mg/g) depending on the annealing time are presented in Figure 11b. Data are presented as the mean of three different samples with the standard deviations. As can be seen from the presented data, the As(III) ions sorption capacity of the studied samples varies nonlinearly; the *Q*e value gradually increases to 48.7% in the first 10 h of annealing compared to unannealed sample, then a 24% reduction in *Q*e occurs when the annealing time reaches 24 h. As in the case of catalytic activity, we assume that the decrease in sorption capacity can be attributed to the change in the DC value of copper(II) oxide MTs in the composite content. The increase in crystallinity lowers porosity and thus surface area, which leads to a surface where the amount of functional groups and defects known to be energetic sites in sorption reduces. Therefore, a higher degree of crystallinity leads to less sorption capacity. The mechanism of the As(III) sorption strongly depends on the nature of the interactions between adsorbent and adsorbate [111]. According to previous studies [33,104], the adsorption process of As(III) involves oxidation of As(III) to As(V) by the surface of Cu/CuO TeMs, and then reduction of CuO to Cu_2_O as verified in [112].

A comparison of maximum As(III) sorption capacities of different adsorbents with those attained in this work is presented in Table 4. The results in this table clearly show that very competitive results compared to existing alternatives are achieved in terms of removal of As(III) ions form the aqueous media.

## 4. Conclusions

This paper presented the results of thermal annealing process of PET-based composite track-etched membranes with embedded copper microtubules. After analysis of data on the phase composition and degree of crystallinity of microtubules before and after annealing, it was found that copper was completely converted into copper(II) oxide at 140 °C and an annealing time of more than 10 h.

For the assessment of the catalytic activity of synthesized Cu/CuO/PET composites, liquid-phase hydrogenation reactions of some nitroaromatic compounds were studied. It was found that with an increase in annealing time and thus CuO tenorite phase ratio, the catalytic activity of composites increased significantly and the samples exhibited maximum activity after 5 h of annealing, where the ratio of CuO phase and the degree of crystallinity were 64.3% and 62.7%, respectively. The reaction time was significantly reduced by more than 60% compared to the unannealed sample. For all three reactions studied, the minimum activation energy was obtained after 5 h of annealing. Examination of the reduction of 4-nitroaniline showed that the catalytic activity of the annealed composite membranes (140 °C, 5 h) remains practically unchanged for five consecutive test cycles.

The composites annealed at 140 °C were also tested in terms of their arsenic(III) ions sorption capacities in flow mode. The sorption capacity of composite membranes annealed over 10 h has been shown to increase by 48.7% compared to the unannealed sample. Cu/CuO/PET membrane composites that successfully combine mechanical strength, possibility of repeated use, low cost, and ease of production can be considered as promising materials for sorption of arsenic ions from aqueous solutions. Moreover, the low-temperature annealing method can be evaluated as an efficient way to increase the catalyst and sorption properties of composite track-etched membranes based on copper microtubules.

## Figures and Tables

**Table 1:** Changes in the crystal structure of the composites according to XRD data after annealing at 140 °C.

Annealing Time, h	Phase	hkl *^a^*	2*θ*°	*d ^b^*, Å	*L ^c^*, nm	*a ^d^*, Å	DC *^e^*, %	FWHM *^f^*	Phase Ratio, %
0	Cu	111	43.48	2.08	9.87	3.592	59.5	0.96	72.5
	CuO	111	36.84	2.44	13.67	4.241		0.68	27.5
3	Cu	111	43.41	2.08	11.71	3.595	60.8	0.81	61.4
	CuO	111	36.47	2.46	8.50	4.255		1.09	38.6
5	Cu	111	43.33	2.09	12.89	3.613	62.7	0.74	35.7
	CuO	111	36.84	2.44	8.40	4.236		1.11	64.3
7	Cu	111	43.26	2.09	18.33	3.627	61.0	0.52	30.9
	CuO	111	36.55	2.46	7.65	4.238		1.22	69.1
10	CuO	111	36.47	2.46	4.99	4.229	41.9	1.86	~100
16	CuO	111	36.77	2.44	6.91	4.235	30.5	1.35	~100
24	CuO	111	36.47	2.46	9.50	4.246	29.7	0.98	~100

*^a^* Miller indices for corresponding planes, *^b^* spacing between planes, *^c^* average crystallite size, *^d^* crystal lattice parameter, *^e^* degree of crystallinity, *^f^* full width at half maximum.

**Table 2:** Change in the *E*a (kJ/mol) value as a function of annealing time and CuO phase ratio as obtained from XRD data.

Decomposed Pollutant Reacted	Annealing Time, h/CuO Ratio From XRD, %			
	0/27.5	5/64.3	10/100	24/100
4-NP	36.97	39.95	26.90	37.92
4-NA	42.55	13.39	21.86	22.18
4-NBA	56.18	52.30	34.82	25.25

**Table 3:** Catalytic activity of the copper and copper(II) oxide nanostructures as well as TeMs-supported catalysts in the hydrogenation reaction of aromatic nitro compounds.

Loaded Catalyst		Studied Aromatic Nitro Compound	Nanocatalyst Efficiency			Ref.
Type	Amount		k, min^−1^	Ea, kJ/mol	D, %	
0.25 wt% Cu/water-washed coal fly ash	400 mg/L	4-NP	0.41	-	-	[90]
Cu NPs (9 nm)	0.0017 mg/L	4-NP	0.26	45.8	-	[91]
CuO NPs	1 mol % CuO	4-NP	-	-	96.0	[92]
Cu NPs	10 mol % Cu	4-NP	-	-	66.0	[93]
		4-NBA	-	-	92.0	
CuO nano/microparticles	5.0 mg/L	4-NP	0.21	-	-	[94]
CuO/γ-Al_2_O_3_ NPs	50 mg	4-NP	0.17	27.4	-	[67]
Cu/PET TeMs	3.2 mg	4-NP	0.52	28.3	83.8	[30]
Cu/CuO/PET TeMs (5 h, 140 °C)	3.2 mg (2 × 2 cm)	4-NP	0.29	39.9	98.1	This study
		4-NA	0.26	13.4	90.6	
		4-NBA	0.30	52.3	83.9	

**Table 4:** As(III) adsorption capacities of various nanosized adsorbents.

Adsorbent	Testing Mode	mg As(III)/g Adsorbent	Ref.
CuO NPs	Batch	26.9	[33]
Cu/Carbon NT membrane	Cross-flow	45.0	[36]
Cu/CuO/PET TeMs (10 h, 140 °C)	Cross-flow	71.7	This study
Cu/PET TeMs (unannealed)	Cross-flow	36.7	

## Supplementary Materials

The following are available online at https://www.mdpi.com/2079-4991/10/8/1552/s1, Figure S1: TGA and DTG curves of the PET template; Figure S2: Pseudo-first-order kinetics for hydrogenation of 4-NP, 4-NA, and 4-NBA in the presence of the Cu/CuO composite TeMs; Figure S3: The Arrhenius plots of ln k vs. 1/T for the reduction reaction of 4-NP, 4-NA, and 4-NBA with NaBH_4_ at different temperatures in the presence of Cu/CuO composite TeMs.
