# Supplementary material for: Cu/CuO Composite Track-Etched Membranes for Catalytic Decomposition of Nitrophenols and Removal of As(III)
Source: Nanomaterials (Basel). 2020 Aug 7;10(8):1552. doi: 10.3390/nano10081552 (PMC7466412; doi:10.3390/nano10081552)
Supplement: Supplementary file 1 [file nanomaterials-10-01552-s001.pdf]

nanomaterials

MDPI

Supplementary Materials

Cu/CuO Composite Track-Etched Membranes for Catalytic Decomposition of Nitrophenols and Removal of As(III)

Anastassiya A. Mashentseva [1,2,*], Murat Barsbay [3], Maxim V. Zdorovets [1,2,4], Dmitriy A. Zheltov [1] and Olgun Güven [3]

[1] The Institute of Nuclear Physics of the Republic of Kazakhstan, Ibragimov str., 1, 050032 Almaty, Kazakhstan; mzdorovets@gmail.com (M.V.Z.); zheltovda@gmail.com (D.A.Z.)
[2] Engineering Profile Laboratory, L.N. Gumilyov Eurasian National University, Satpaev str., 5, 010008 Nur-Sultan, Kazakhstan
[3] Department of Chemistry, Hacettepe University, 06800 Ankara, Turkey; mbarsbay@hacettepe.edu.tr (M.B.); guven@hacettepe.edu.tr (O.G.)
[4] Department of Intelligent Information Technologies, Ural Federal University named after the first President of Russia B. N. Yeltsin, Mira str. 19, 620002 Yekaterinburg, Russia
* Correspondence: a.mashentseva@inp.kz or mashentseva.a@gmail.com

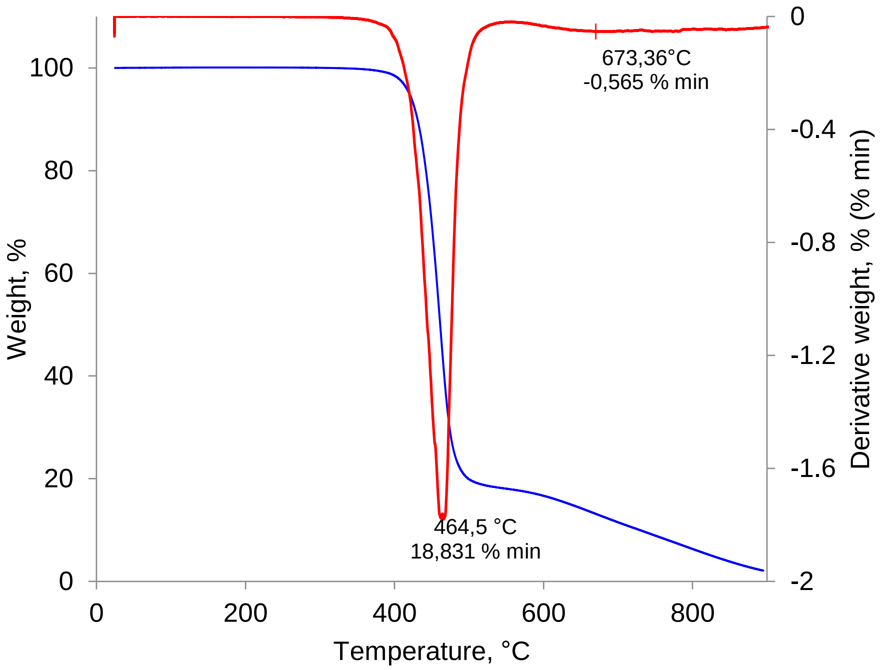

Figure S1. TGA and DTG curves of the PET template.

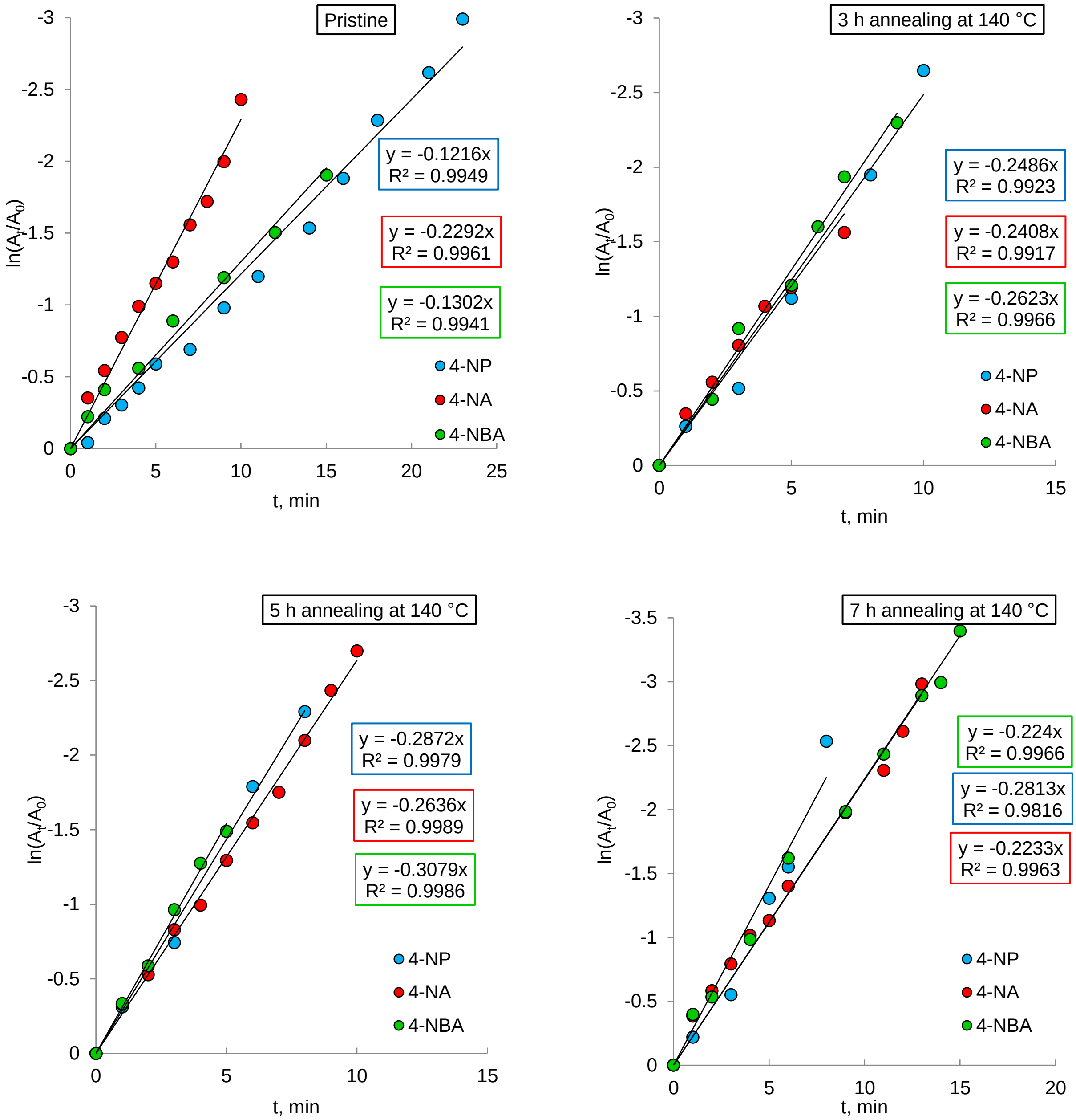
Pristine
y = -0.1216x
R² = 0.9949
y = -0.2292x
R² = 0.9961
y = -0.1302x
R² = 0.9941
4-NP
4-NA
4-NBA
ln(At/A0)
t, min
3 h annealing at 140 °C
y = -0.2486x
R² = 0.9923
y = -0.2408x
R² = 0.9917
y = -0.2623x
R² = 0.9966
4-NP
4-NA
4-NBA
ln(At/A0)
t, min
5 h annealing at 140 °C
y = -0.2872x
R² = 0.9979
y = -0.2636x
R² = 0.9989
y = -0.3079x
R² = 0.9986
4-NP
4-NA
4-NBA
ln(At/A0)
t, min
7 h annealing at 140 °C
y = -0.224x
R² = 0.9966
y = -0.2813x
R² = 0.9816
y = -0.2233x
R² = 0.9963
4-NP
4-NA
4-NBA
ln(At/A0)
t, min

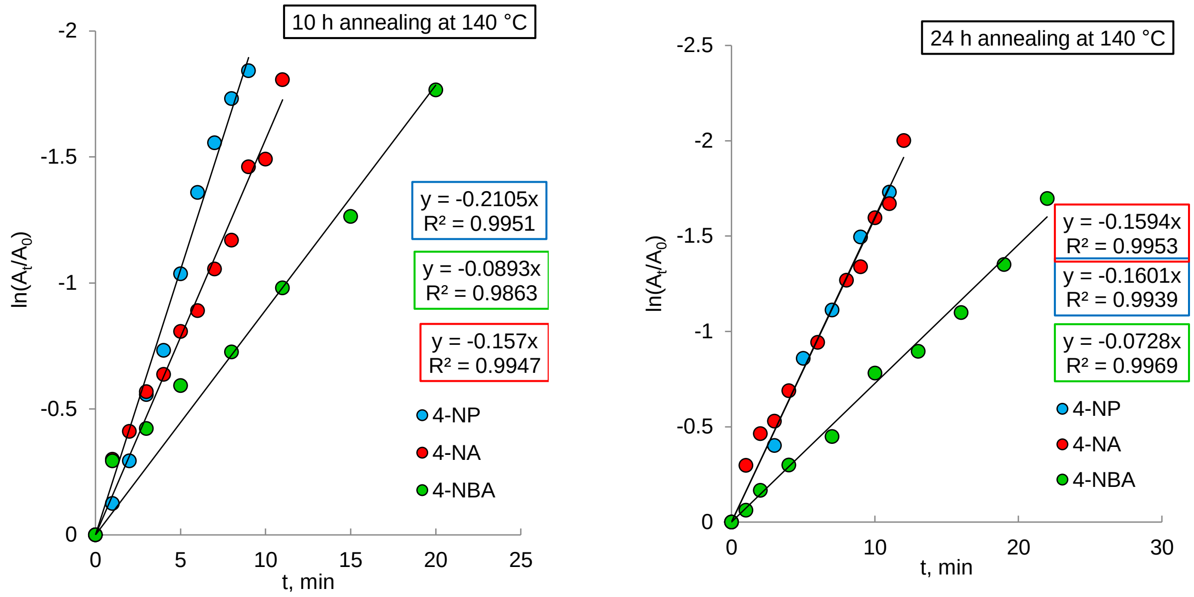

Figure S2. Pseudo-first-order kinetics for hydrogenation of 4-NP, 4-NA and 4-NBA in the presence of the Cu/CuO composite TeMs.

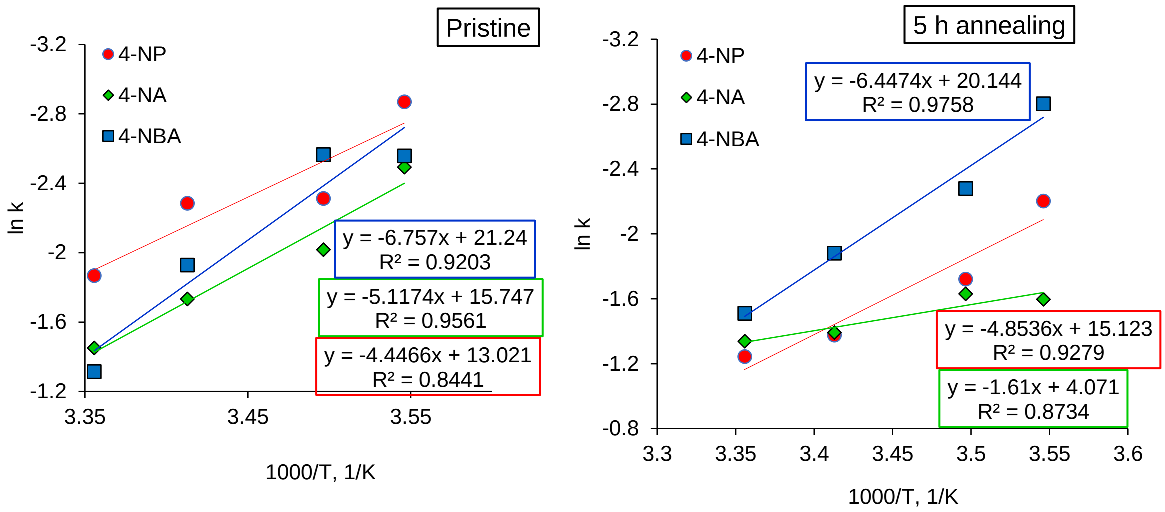

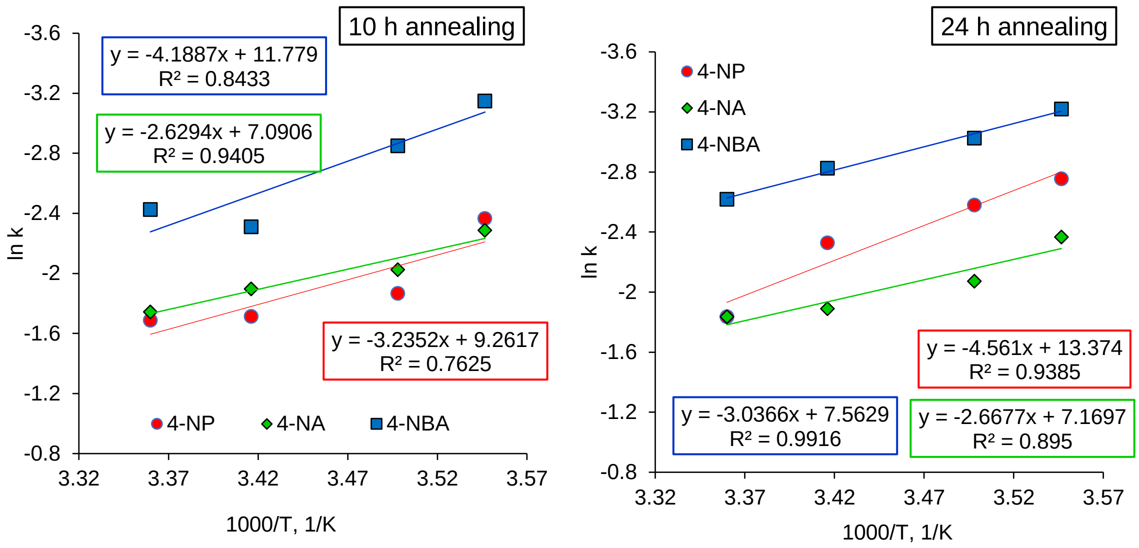

Figure S3. The Arrhenius plots of ln k vs. 1/T for the reduction reaction of 4-NP, 4-NA and 4-NBA with NaBH4 at different temperatures in the presence of Cu/CuO composite TeMs.
